# Phytochemical Screening and Antioxidant Activity of Selected Estonian *Galium* Species

**DOI:** 10.3390/molecules28062867

**Published:** 2023-03-22

**Authors:** Pille-Riin Laanet, Piret Saar-Reismaa, Piia Jõul, Olga Bragina, Merike Vaher

**Affiliations:** 1 Department of Chemistry and Biotechnology, Tallinn University of Technology, Akadeemia tee 15, 12618 Tallinn, Estonia; 2 HAN BioCentre, Laan van Scheut 2, 6525 EM Nijmegen, The Netherlands; 3 National Institute for Health Development, Hiiu 42, 11619 Tallinn, Estonia

**Keywords:** phytochemicals, plant extracts, polyphenols, iridoids, antioxidants, *Galium verum*, *Galium aparine*, *Galium mollugo*, green chemistry, SPME

## Abstract

The aim of the present study was to examine three different *Galium* species from the native population of Estonia, *Galium verum*, *Galium aparine*, and *Galium mollugo*, to characterise their non-volatile and volatile phytochemical composition and antioxidant activity. The main groups of bioactive compounds in the plants were quantified by colorimetric tests, showing high concentrations of polyphenols (up to 27.2 ± 1.5 mg GAE/g), flavonoids (up to 7.3 ± 0.5 mg QE/g) and iridoids (up to 40.8 ± 2.9 mg AE/g). The species were compared using HPLC-DAD-MS/MS, revealing some key differences in the phytochemical makeup of the extracts. The most abundant compound in the extracts of *Galium verum* blossoms and herb was found to be asperuloside, in *Galium aparine* herb, asperulosidic acid, and in *Galium mollugo* herb, chlorogenic acid. Additionally, the composition of volatile compounds was analysed by SPME-GC-MS. The degree of variability between the samples was high, but three volatiles, hexanal, anethole, and *β*-caryophyllene, were quantified (≥1%) in all analysed samples. The antioxidative activity of all extracts was evaluated using the ORAC_FL_ method, demonstrating that the *Galium* species from Estonia all exhibit strong antioxidant capacity (up to 9.3 ± 1.2 mg TE/g). Out of the extracts studied, *Galium verum* blossoms contained the highest amounts of bioactives and had the strongest antioxidant capacity.

## 1. Introduction

Ethnomedicine has long used different plants for a variety of disease preventative and therapeutic purposes. Plants are a natural source of active compounds, phytochemicals, many of which with favourable bioactivity for humans. Analytical investigation allows for the determination of the phytochemical composition and confirmation of specific therapeutic properties of the plants already acknowledged for their potential by ethnomedicine [1,2,3,4,5]. The *Galium* L. genus, comprising about 667 species found worldwide, over a third of which can be found in Europe, includes several species that have been used in traditional medicine to alleviate a variety of ailments [6,7]. *Galium verum* has been used as a sedative, diuretic, and choleretic, as well as to treat gout, epilepsy, and spasms; *Galium aparine* has been used to reduce infection and inflammation, to treat wounds, burns, and skin diseases; *Galium mollugo* has been used as vulnerary, and to treat hysteria and epilepsy [7,8,9]. Many scientific studies have confirmed that the representatives of the *Galium* genus exhibit a wide range of biological activities, including antioxidant, anticancer, detoxicant, hepatoprotective, antihaemolytic, antibacterial, and antifungal effects [2,7,9,10,11]. Phytochemicals are typically extracted from plant materials using alcohols or acetone in varying proportions of water, depending on the properties of the compounds of interest [9]. Therefore, a comparative analysis of the earlier literature is complicated due to the use of varying extraction solvents. The most common analysis methods of plant extracts are HPLC or UHPLC, coupled with DAD and/or MS/MS, sometimes followed by identity confirmation with NMR [9,12,13]. Previously, Mitova et al. have analysed the iridoid patterns of the Bulgarian *Galium* species [12], and Vlase et al. have studied the polyphenolic content of Romanian *Galium* species [13]. As it is known that the geographic origin and growth environment of a plant play a large role in the phytochemical content of the extract [14], it is likely that the *Galium* species collected from different locations in Europe exhibit a different phytochemical profile. Therefore, it is important to assess the constituents of all medicinal plants in relation to their growth habitat. To the best of our knowledge, species of *Galium* native to a northern European country have not yet been thoroughly characterised. Additionally, when evaluating plant extracts as possible therapeutic agents, the assessment of antioxidative capacity is of clear importance. As excess free radicals are known to damage cellular components and thereby be involved in the development of several diseases, the free radical scavenging properties of antioxidants can alleviate or prevent illness [15].

Studies of medicinal plants have demonstrated that the health-promoting properties and therapeutically beneficial qualities can often be attributed to specific polyphenols or iridoids, two of the most prevalent groups of bioactives in many plant extracts, and in the representatives of the *Galium* genus [9,11,16,17]. Polyphenols are the most abundant secondary metabolites in plants, notably characterised by their potent antioxidant properties [18]. Polyphenols also modulate the activity of many enzyme and cell receptors, scavenge free radicals, regulate nitric oxide, decrease leukocyte immobilization, induce apoptosis, and inhibit cell proliferation and angiogenesis [18,19,20,21,22,23]. Therefore, polyphenols possess several mechanisms for preventing and treating illnesses. As deduced from epidemiologic data, polyphenols exert preventative effects over cardiovascular, neurodegenerative, and oxidative stress associated diseases, such as cancer [19,24,25,26,27]. Plant phenolics include phenolic acids, flavonoids, tannins, stilbenes and lignans. Phenolic acids include derivatives of benzoic acid such as gallic acid, and derivatives of cinnamic acid, such as coumaric, caffeic and ferulic acid. Flavonoids are the most prevalent polyphenols in the human diet [18], and possess a variety of pharmacological effects mainly tied to their free radical scavenging and antioxidative properties, such as hepatoprotective, antiatherosclerotic, anti-inflammatory, antithrombogenic, antitumor, antiosteoporotic, antibacterial, and antiviral effects [22,28,29,30]. Some of the most common flavonoids include quercetin, catechin, naringenin, kaempferol, rutin, cyanidin-glycoside, daidzein, genistein, and glycitein [19,31]. Prevalent in all land plants, the second major group of active compounds are iridoids, mainly found as conjugates, iridoid glycosides [32]. Derivatives of iridoids, secoiridoids, are present in about 57 plant families [33]. Specific iridoids can be used as chemical markers of several genera in different plant families, for example, asperuloside of *Galium*, aucubin of *Plantago*, and aucubin and harpagide of *Scrophularia* [12,34]. Iridoids are found in numerous ethnomedicinal plants that have been used as sedatives, hypotensives, antipyretics, and to treat diabetes, cough, wounds, skin disorders, and other inflammatory diseases [33,35,36]. Pharmacological research has confirmed that naturally occurring iridoids exhibit a variety of useful functionalities: neuroprotective, immunomodulatory, antidiabetic, cardioprotective, antihepatotoxic, hepatoprotective, choleretic, hypoglycemic and hypolipidemic, anti-inflammatory, wound healing, antispasmodic, antitumor, antiviral, antibacterial, and antifungal activities [32,33,36,37,38,39,40].

In addition to non-volatile compounds, volatile phytochemicals are also known to possess many favourable biological activities, including antimicrobial, antioxidant, and anti-inflammatory effects [41,42]. There are a few studies on the comparison of chemical volatile profiles of *Galium* species [43,44,45,46]. Ciotlaus et al. analysed volatile compounds in *Galium verum* by SPME-GC-MS method using Carboxen/PDMS fibre coating and compared fresh and dried plant material [43]. The major compounds identified in the floral bouquet of dried flower were hexanal, Z-2-hexenal, 1-hexanol, eucalyptol, linalool, and camphor [43]. According to the Green Analytical Chemistry and Green Sample Preparation principles, SPME-GC-MS analysis procedure should be preferable compared to traditionally used essential oil extraction methods, such as steam- or hydrodistillation paired with GC-FID analysis [47,48,49]. The SPME-GC-MS analysis procedure is more sensitive, consumes less energy, involves no solvent use, and minimizes sample volume and waste production, but allows for a comparison of the composition of volatile compounds in different plant species [50]. The PDMS/DVB SPME fibre is coated with a mixed (bipolar) phase, which allows the analysis of both more polar and nonpolar compounds from the headspace and is often used for the analysis of volatiles from plant material [50].

The aim of this paper was to examine the hydroalcoholic and -acetonic extracts of the native Estonian *Galium* species (*Galium verum* blossoms, *Galium verum* herb, *Galium aparine* herb, *Galium mollugo* herb) to determine their non-volatile phytochemical profile and antioxidant potential dependent on the three different extraction solvents used. Moreover, a thorough profiling of the volatiles found in these plants helps to widen the reach of the potential therapeutic applications for the Estonian *Galium* species.

## 2. Results and Discussion

### 2.1. Colorimetric Assays and Antioxidativity

The antioxidant activities and the total polyphenolic, flavonoid, and iridoid content of the extracts are shown in Table 1. The Estonian *Galium* species, especially *Galium verum*, are an excellent source of polyphenols and iridoids. As discussed earlier, this shows the great therapeutic potential of these plants. The results suggest that extraction with 50% acetone yields the highest quantities of polyphenols (up to 25.1 ± 0.8 mg GAE/g) and flavonoids (up to 7.3 ± 0.5 mg QE/g), whereas 50% ethanol should be used to obtain extracts containing large amounts of iridoids (up to 36.6 ± 2.2 mg AE/g). Across the different extracts, both 50% acetone and 50% ethanol extracts are characterised by high antioxidant activities (up to 9.3 ± 1.2 and 7.2 ± 1.4 mg TE/g, respectively). The colorimetric analyses demonstrated that the extracts of *Galium verum* blossoms generally contained the highest amounts of bioactives and had the strongest antioxidant properties. Additionally, the results from these experiments exemplify the linear correlation between the total polyphenolic content and the antioxidativity of the *Galium* extracts (correlation graph provided in Supplementary Materials, Figure S1), illustrated by the two extracts with the highest polyphenolic content also having the strongest antioxidative properties (namely, the 50% acetone extracts of *Galium verum* blossoms and *Galium mollugo* herb). Therefore, our results confirm the earlier reports of antioxidant properties being attributed to the polyphenolics the extracts contain. Regardless of the extraction solvent used, both *Galium verum* blossoms and *Galium mollugo* herb showed high polyphenolic content and strong antioxidant properties.

### 2.2. Identification and Quantification of Major Constituents in Extracts

**Figure 1:**
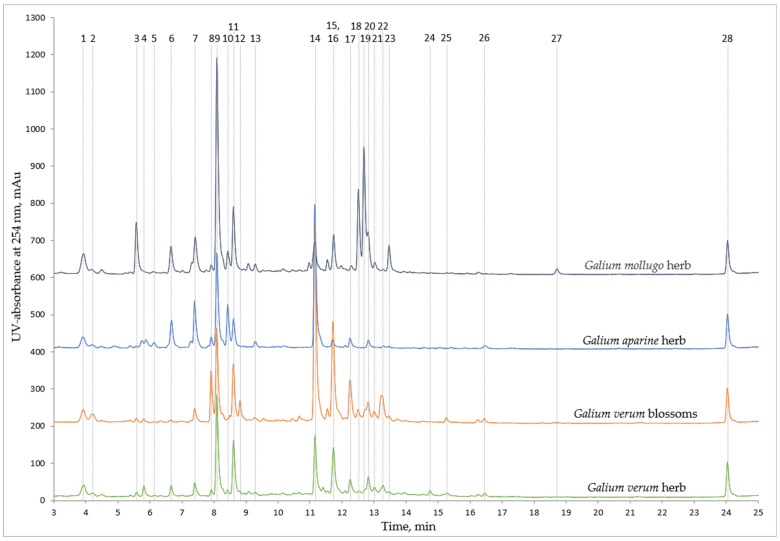
All quantified phytochemicals on chromatograms of 50% ethanol extracts of analysed *Galium* species.

The 27 non-volatile compounds that were quantified (Figure 1, Table 2 and Table 3) were chosen as the most representative to characterise both the similarities and differences amongst the *Galium* species. The results indicate a few key characteristics that clearly differentiate the chromatographic profile and the phytochemical content of the extracts. The specific phytochemicals in the analysed extracts were identified using reference standards and HPLC-DAD-MS/MS results, considering the UV spectra, molecular ion, and fragmentation patterns in comparison with the literature (Figure 1, Table 2). The phytochemicals were then quantified as described in the Materials and Methods section, according to the DAD signal and standard compound calibration curves (Table 3). The phytochemicals that were identified and quantified in all extracts were deacetylasperulosidic acid, asperulosidic acid, chlorogenic acid, asperuloside, rutin, and quercetin (compounds 1, 7, 9, 11, 14, 26). Neochlorogenic acid (comp. 6) was quantified in all extracts but the ones of *Galium verum* blossoms, showing the highest concentrations in *Galium mollugo* extracts. Additionally, the high content of diosmetin isomers (comp. 18, 19, 23) in *Galium mollugo* clearly sets apart this chromatographic profile from others. *Galium aparine* extracts are characterised by a comparatively large cryptochlorogenic acid (comp. 10) peak and a reduced asperuloside (comp. 11) peak, separating the profile of these extracts from other plants. *Galium mollugo* extracts also contain a significant amount of cryptochlorogenic acid (comp. 10), but the asperuloside (comp. 11) peak is similar in size to the extracts of both *Galium verum* blossoms and herb. The extracts of *Galium verum* blossoms contained significantly higher amounts of rutin (comp. 14) than other extracts and were also characterised by the largest isorhamnetin-3-O-rutinoside (comp. 17) peak. Whereas the general profile of the *Galium verum* herb extracts are like the ones of the *Galium verum* blossoms, the phytochemical content and the chromatographic peak sizes are lesser in the former. Aside from the great difference in the size of the rutin (comp. 14) peak, the appearance of the quercetin-3-rutinoside-7-glucoside (comp. 8) peak in the *Galium verum* blossoms extract also differentiates the chromatographic profiles of the two extracts of *Galium verum*. It is therefore possible to differentiate between the different species of *Galium* based on the general profile of HPLC-DAD analyses.

The study of the Bulgarian *Galium* species conducted by Mitova et al. revealed that *Galium verum* and *mollugo* share a similar phenolic profile, one of the similarities being the involvement of diosmetin. Even though this could be stated to be true for Estonian species, the abundance of diosmetin isomers in our extracts distinctly differentiates the two chromatographic profiles as the corresponding peaks are much lesser in size for *Galium verum* (Figure 1). Mitova et al. also analysed the iridoid patterns of the Bulgarian *Galium* species and found that *Galium verum, aparine* and *mollugo* all contain asperulosidic acid [12], and this was confirmed by our data to also be true for the Estonian species (comp. 7). When studying the polyphenolic content of 70% ethanol extracts of Romanian *Galium* species, Vlase et al. found that *Galim verum, aparine* and *mollugo* all contained chlorogenic acid, rutin, and quercetin [13]. Our research demonstrates that 50% acetone, 50% ethanol, and 80% ethanol extracts of these species all contain these polyphenols also (comp. 9, 14, 26). It is important to note that the abundance of these polyphenolics between the different species is varied, and, in general, the use of a less polar solvent (50% acetone) helps enhance the quantities of polyphenolics in the extracts (Table 2). Interestingly, kaempferol-7-O-glucoside (comp. 16) was identified in both *Galium verum* and *Galium mollugo*, whereas in the study of Bulgarian *Galium* species by Mitova et al., only *Galium verum* was shown to contain kaempferol glycosides [12]. This finding demonstrates how the phytochemical makeup of a species can vary based on the geographic origin.

Our research also demonstrates the differences in phytochemical content dependant on the extraction solvent used (and its polarity), as 50% acetone, 50% ethanol, and 80% ethanol extracts showed varied yields of phytochemicals quantified (Table 3). The extracts of *Galium verum* blossoms contained the highest amounts of the same two compounds, asperuloside and rutin, regardless of the extraction solvent used. Thirdly, either dicaffeoylquinic acid isomer (comp. 22; in the 50% acetone and 80% ethanol extract), or chlorogenic acid (in the 50% ethanol extract), was found. In the *Galium verum* herb extract, the phytochemical in the highest abundance was also asperuloside, followed by chlorogenic acid, and thirdly, either deacetylasperulosidic acid (50% ethanol extract) or asperulosidic acid (50% acetone and 80% ethanol extracts). In *Galium aparine* extracts, the most abundant phytochemical was either asperulosidic acid (50% acetone and 50% ethanol extracts) or asperuloside (80% ethanol extract). The second and third compound were similar in hydroethanolic extracts, chlorogenic acid and rutin, and differed in the hydroacetonic extract, rutin and asperuloside. Finally, in the *Galium mollugo* extracts, the three most common phytochemicals were similar, but found in differing amounts for each extraction solvent: these compounds were chlorogenic acid (in highest abundance in 50% acetone); asperuloside (in highest abundance in 80% ethanol); and diosmetin isomer, comp. 19 (in highest abundance in 50% ethanol). In conclusion, the most abundant compound in the extracts of *Galium verum* blossoms and herb was found to be asperuloside, in *Galium aparine* herb, asperulosidic acid, and in *Galium mollugo* herb, chlorogenic acid. Asperuloside has shown to have a wide array of therapeutically beneficial qualities [51]. Hence, it is important to note that the species with the highest abundance of asperuloside is *Galium verum*, with the extracts of *Galium verum* blossoms showing higher amounts of asperuloside compared to the extracts of the whole aerial part of the plant. Additionally, a less polar solvent should be preferred when extracting asperuloside from *Galium* species, as the yields were shown to be more favourable with hydroacetonic extracts compared to the more polar, hydroethanolic extracts. On the other hand, it is noteworthy that the yields were not significantly less in the hydroethanolic extracts. This is an advantage for future biological applications, as acetonic extracts would not be suitable for such purposes. Extracts of Estonian *Galium* species are also rich in polyphenolics, especially chlorogenic acid and rutin. As polyphenols are strong antioxidants, these extracts could also be used in therapeutic research against oxidative stress induced illnesses.

### 2.3. Analysis of Volatiles by SPME-GC-MS

The volatiles of *Galium verum* blossoms, herb, *Galium aparine* herb, and *Galium mollugo* herb were quantified and are shown in Table 4, which includes only the compounds that amount to more than 1.0% of total volatiles in each sample. Significant quantitative and qualitative differences were observed between the different *Galium* species. There were three volatile compounds, hexanal, anethole, *β*-caryophyllene, that were identified in all analysed samples (out of all compounds that made up more than 1.0% of total volatiles in a specific sample). According to the previous studies, these are all compounds that should be present in *Galium* species [43,44,45,46]. It has previously been proven that anethole is effective against yeast, bacterial and fungal strains [52,53], and caryophyllene-rich oil has been reported to have high inhibitory activity against some fungi and bacteria [54].

In general, the volatiles in *Galium verum* blossom and herb samples were similar. However, the herb sample contained a larger variety of volatile compounds, for example, *β*-terpinen, *β*-pinene, copaene, linalool, estragole and dihydroactinidiolide did not occur in the blossom sample.

Table 4 gives a comprehensive overview of the volatile compounds that were identified in the different *Galium* species and demonstrates the high diversity present in the chemical composition of the samples. The highly variable content of volatile compounds certainly contributes to the potential and widespread use of these species, e.g., in the cosmetics and pharmaceutical industry.

## 3. Materials and Methods

### 3.1. Plant Material and Preparation of Plant Extracts

*Galium verum* herb was purchased from Kubja Ürt OÜ (Tallinn, Estonia). *Galium verum* blossoms were purchased from Norman Ravimtaimed OÜ (Karepa, Estonia). *Galium aparine* and *Galium mollugo* herbs were obtained from a private garden in Saaremaa, Estonia. All the plant materials studied were previously air-dried and kept at an ambient temperature in a dark space before being subjected to the extraction procedure.

The plant material was ground finely using a coffee bean grinder Bomann KSW 445 CB (China). The solvent used for the extraction of plant material was either 50% ethanol, 80% ethanol, or 50% acetone (*v*/*v* in ultrapure water), the ratio of plant material to solvent 1:20 (*w*/*v*). The procedure included 30 min of shaking using OrbitalShaker DOS-20M (Latvia) at 250 r/min (with a direction change after 99 r), then sonication at 640 W (350 kHz) in Sonorex™ Digital 10P bath (Bandelin, Germany) for 30 min at 35 °C. The extract was then vacuum filtered through a Sartorius^TM^ 3 h filter (70 mm, 65 g/m^3^) (Sartorius, France). All the following qualitative and quantitative analyses were performed in triplicate for all the four different plant samples using three different extraction solvents, for each replicate the extract was prepared by a different member of the research group.

### 3.2. Chemicals

Ultrapure water (≥18 MΩcm) produced within the laboratory with a Milli-Q water purification system (Merck KGaA, Darmstadt, Germany) was used to prepare all aqueous solutions. Extraction solvent ethanol (96.7%) was obtained from Sigma-Aldrich (Munich, Germany). Acetonitrile (≥99.9%), an HPLC eluent, and acetone (≥99.9%), an extraction solvent, were purchased from Honeywell (Seelze, Germany). Formic acid (≥99.0%) used in HPLC eluents was purchased from Fisher Chemical (Czech Republic). Total polyphenolic content of the extracts was evaluated using the 2 M Folin–Ciocalteu reagent, purchased from Sigma-Aldrich (Switzerland), and water-free sodium carbonate, purchased from Sigma-Aldrich (Germany). Standard solutions used were prepared from gallic acid monohydrate (Sigma-Aldrich, China) and 96.7% ethanol (Sigma-Aldrich, Germany). For the detection of total flavonoid content, aluminium chloride, obtained from Fluka (Switzerland), was used. Standard solutions were prepared from quercetin (≥99.0%, Lachema/Chemapol) and methanol (≥99.9%, Honeywell, Charlotte, NC, USA). Total iridoid content was determined using the Trim–Hill reagent, prepared from water-free acetic acid (≥99.0%, Sigma-Aldrich, Germany), 37% hydrochloric acid (Honeywell/Fluka, Austria), and copper sulfate pentahydrate (Sigma-Aldrich, Munich, Germany). Standard solutions were prepared using asperuloside purchased from MedChemExpress. Fluorescein sodium salt (≥98.5%) and AAPH (2,2′-azobis(2-methyl-propionamidine)dihydrochloride, 97%) used in the antioxidativity studies, were purchased from Fluka (Switzerland) and Sigma-Aldrich (Germany), respectively. Standard solutions were prepared from Trolox (6-hydroxy-2,5,7,8-tetramethylchroman-2-carboxylic acid, 97%), purchased from Sigma-Aldrich (Germany). Standard compounds used for phytochemical quantification, asperuloside (≥95%), neochlorogenic acid (≥98%), chlorogenic acid (≥95%), rutin (≥95%), and quercetin (≥95%), and internal standard, bicalutamide (≥99.8%), were all purchased from Sigma-Aldrich (Germany).

### 3.3. Colorimetric Analyses

The main groups of bioactive compounds in the plants were quantified by colorimetric tests: total flavonoids by the AlCl_3_, total iridoids by the Trim–Hill, and total polyphenols by the Folin–Ciocalteu method [55,56,57]. All colorimetric analyses were conducted on the Varian Cary 50 Bio UV-Vis spectrophotometer (Agilent Technologies, Santa Clara, CO, USA), using 1.5 mL single-use semi-micro cuvettes (Nerbe Plus GmbH & Co. KG, Winsen (Luhe), Germany). Total polyphenolic content of the extracts was determined using calibration solutions of 10, 25, 50, 75, and 100 mg/L of gallic acid in ethanol, prepared from a 5 g/L stock solution. The calibration solutions for the quantification of flavonoid contents were prepared in concentrations of 2, 5, 10, 20, and 40 mg/L of quercetin in methanol, prepared from a 2 g/L stock solution. The calibration solutions for iridoid content measurements were prepared from a 1 g/L stock solution of asperuloside in a solvent corresponding to the extraction solvent. The concentrations for calibration solutions were 100, 200, 400, 800, and 1000 mg/L. All samples were measured in triplicate and the results given in mg of either mean gallic acid, quercetin, or asperuloside equivalents per g of plant material (mg GAE/QE/AE/g) ± standard deviation (Table 1).

### 3.4. Evaluation of Antioxidative Properties

The antioxidative activity of all extracts was evaluated using the ORAC_FL_ (oxygen radical absorbance capacity) method with minor modifications as described by Naguib [58]. The 24.25 mM fluorescein stock solution, 30 mM Trolox stock solution, and 600 mM AAPH solution were all prepared daily in a 100 mM phosphate buffer (pH = 7.4). The total volume of the reaction mixture was 3 mL, the mixture was composed of 2.7 mL of 24.25 nM fluorescein and 100 µL of sample/Trolox dilution, which was incubated at 37 °C for 3 min, and then 200 µL of 600 mM AAPH was added. The samples were measured by a Hitachi F-7000 Fluorescence Spectrophotometer (Chiyoda, Tokyo, Japan) at λex/em 495/520 nm, slits 5 nm, and the time scan was recorded for 3000 s once per s. The calibration solutions were prepared from a diluted, 300 µM Trolox solution, in concentrations of 0.5, 1, 2, 4, 6, 8, and 10 µM, and the calibration was given as area under curve change from 0 µM Trolox (blank sample). All extracts were analysed diluted 500-fold. Due to the matrix effects apparent in the diluted samples of 80% ethanol extracts, the calibration samples were prepared in 80% ethanol diluted 500-fold in phosphate buffer. For 50% ethanol and 50% acetone extracts, the matrix effects were negligible (<5%), and the Trolox calibration samples were prepared in phosphate buffer. All samples were measured in triplicate and the results given in mg of mean Trolox equivalents per g of plant material (mg TE/g) ± standard deviation (Table 1).

### 3.5. Phytochemical Screening and Quantification

The 1 mg/mL standard solutions of asperuloside, neochlorogenic acid, chlorogenic acid, rutin, and quercetin were prepared in ethanol, and diluted to create calibration curves for the quantitative HPLC-DAD analyses. The calibration curves were constructed based on the UV signal at 254 nm (slit 4 nm), using the ratio of the peak area of a standard compound to the peak area of the internal standard bicalutamide (2 g/L ethanolic solution was added at a final concentration of 40 mg/L into all the samples). All standard compounds exhibited a linear range between 5 to 250 mg/L. Other phytochemicals detected on the chromatograms were also quantitatively determined using these calibration curves, by matching these compounds with a standard compound based on the similarities in the respective UV spectra. The DAD spectra were measured from the analyses of 50% ethanol extracts in the range of 200 to 400 nm. These selections and respective results are given in Table 3 (all measured in triplicate and given as mean mg per L of extract ± standard deviation), and all UV spectra as Figures S2–S29 in the Supplementary Materials.

Before the HPLC-DAD-MS/MS analyses, the extracts were centrifuged, the sample then diluted in ultrapure water two-fold, and spiked with the internal standard bicalutamide at a final concentration of 40 mg/L. The sample volume injected was 5 µL. HPLC-DAD-MS/MS analyses were conducted using an Agilent 1260 Infinity II instrument (Agilent Technologies, Inc., USA) with an Agilent Poroshell 120 EC-C18 column, particle size 2.7 µm, measurements 4.6 mm × 100 mm (Agilent Technologies, Inc., USA) thermostated at 28 °C. The mobile phase consisted of ultrapure water (A) and acetonitrile (B), both acidified with 0.1% (*v*/*v*) formic acid. The elution procedure was a linear gradient increasing from 5% to 50% B (0–20 min), then from 50% to 95% B (20–25 min), isocratic 95% B (25–30 min), a linear gradient decreasing from 95% to 5% B (30–30.01 min), and isocratic 5% B (30.01–35 min). The flow rate was kept at 0.6 mL/min. The column was coupled with an Infinity 1260 DAD (Agilent Technologies, Inc., USA), the chromatograms were recorded at a UV absorbance wavelength of 254 nm (slit 4 nm), and DAD spectra in the range of 200 to 400 nm. Following the DAD analysis, the sample was analysed using the LC/MSD Trap XCT mass spectrometer (Agilent Technologies, Inc., USA) equipped with an electrospray ionization source. The mass spectra were recorded in negative-ion mode, in the *m*/*z* range from 100 to 1000. Nitrogen was used as the nebulizing and drying gas, and helium served as the collision gas. The MS/MS fragmentation patterns (generated using the automatic Agilent software MS/MS settings) were used to identify compounds for which there were no standard compounds available.

### 3.6. Analysis of Volatiles by SPME-GC-MS

Headspace solid phase microextraction (SPME) coupled with GC-MS allows to determine and compare volatile compounds composition in plant material. In the current study, the SPME procedure was performed on PDMS/DVB Stabile Flex fiber (polydimethylsiloxane/divinylbenzene coating thickness 65 μm, Supelco, Bellefonte, PA, USA) using a manual SPME fiber holder (Supelco-57330-U). SPME fiber was conditioned according to the manufacturer’s instructions prior to the first use. 50 mg of dried and powdered sample was placed into a 1.5 mL glass vial and closed. The vials were thermostated for 15 min at 50–55 °C to perform headspace extraction of volatiles from plant material. The fiber was then withdrawn from the needle and inserted into the GC injection port, where the analytes were thermally desorbed for the GC-MS analysis.

Chromatographic separations were performed on an Agilent Technologies 7890A GC system equipped with an ultra-inert splitless liner (Agilent Technologies, type 5190-2293). The gas chromatograph was coupled to an Agilent 5975C mass spectrometer with an electron ionization source and a quadrupole mass analyser. The flow rate of carrier gas (helium 6.0, AGA, Estonia) was kept constant at 1.2 mL/min and compounds were separated in a ZB-5plus capillary column (30 m × 0.25 mm × 0.25 μm, Agilent Technologies, USA). The injector temperature was kept at 275 °C, injection was performed in the splitless mode for 2 min. The following oven temperature program was used: the initial temperature was 35 °C, then increased to 200 °C (5 °C min^−1^), and to 280 °C (20 °C min^−1^, held for 2 min). The total run time was 39 min starting from fiber introduction into the injection block. The analyte ionization was performed in electron ionization mode using the electron energy of 70 eV. The interface, ion source, and mass analyser temperatures were set at 280, 230, and 150 °C, respectively. Scan mode in the range of 20–500 *m*/*z* was used for monitoring all analytes. All samples were analysed thrice for confirmation. All the compounds were determined by the National Institute of Standards and Technology 17 (NIST 17) library and Agilent MassHunter Qualitative, Quantitative and Unknowns Analysis were used for data analysis.

## 4. Conclusions

This paper describes the phytochemical studies of three *Galium* species native to northern Europe. Our results show that the selected Estonian *Galium* species (*Galium verum* blossoms and herb, *Galium aparine* herb, and *Galium mollugo* herb) are a valuable source of phenolics with antioxidant properties, iridoids, and volatile phytochemicals. The quantitative studies showed that the most abundant non-volatile compound in the extracts of *Galium verum* blossoms and herb was asperuloside, in *Galium aparine* herb, asperulosidic acid, and in *Galium mollugo* herb, chlorogenic acid. The volatile compounds had a high degree of variability, but three, hexanal, anethole, and *β*-caryophyllene, were quantified (>1.0%) in all analysed samples. Additionally, it was found that some key differences in the chromatographic profiles of the extracts allow for a quick identification of an Estonian *Galium* plant of an unknown species. The *Galium verum* extracts exhibited a comparatively large rutin peak, whereas *Galium aparine* an enhanced cryptochlorogenic acid peak. The prominent peaks of diosmetin isomers set apart the *Galium mollugo* extract from the others. The effects of the polarity of an extraction solvent were characterised by the fact that extraction with 50% acetone yielded extracts rich in polyphenols (up to 25.1 ± 0.8 mg GAE/g) and flavonoids (up to 7.3 ± 0.5 mg QE/g), whereas 50% ethanol was preferable for iridoid extraction (up to 36.6 ± 2.2 mg AE/g). Both 50% ethanol and 50% acetone extracts were characterised by high antioxidant activities (up to 7.2 ± 1.4 and 9.3 ± 1.2 mg TE/g, respectively). As oxidative stress can lead to a variety of severe illnesses, the antioxidative properties of the *Galium* extracts contribute to their potential as future therapeutic agents. Out of the species studied, the *Galium verum* blossoms had the strongest antioxidant properties (up to 9.3 ± 1.2 mg TE/g). The therapeutic potential of several volatile and non-volatile phytochemicals found in the *Galium* species has already been demonstrated. *Galium verum* is rich in asperuloside, which has been shown to have anti-viral, anti-malarial, anti-protozoal, anti-tumorigenic, anti-hypertensive, anti-obesity, immunomodulatory, anti-inflammatory, and antioxidant properties. Estonian *Galium* species are rich in polyphenolics, mainly chlorogenic acid and rutin, known for their antioxidant properties. Volatile phytochemicals anethole and *β*-caryophyllene that were found in all extracts have previously shown potential as antibacterial and antifungal agents. In the future, the extraction procedure could be optimised to fully utilise the Estonian *Galium verum* (especially the blossoms) as a rich source for asperuloside. Though the hydroacetonic extract showed higher yields of asperuloside in the current study, the hydroethanolic yields were not significantly lesser. For potential future purposes, this presents an important advantage, as ethanolic extracts are suitable for biological applications. In conclusion, *Galium* species native to Estonia should be considered for future therapeutic approaches due to their many favourable properties.

## Figures and Tables

**Table 1:** Antioxidant activity, and total polyphenolic, flavonoid, and iridoid content of the *Galium* extracts.

Plant Species	Extraction Solvent	Antioxidant Activity, mg TE/g ^1^	Total Polyphenolic Content, mg GAE/g ^2^	Total Flavonoid Content, mg QE/g ^3^	Total Iridoid Content, mg AE/g ^4^
*Galium verum* blossoms	50% acetone	9.3 ± 1.2	27.2 ± 1.5	7.3 ± 0.5	40.8 ± 2.9
	50% ethanol	7.2 ± 1.4	20.7 ± 1.4	5.6 ± 0.5	36.6 ± 2.2
	80% ethanol	4.3 ± 0.7	14.8 ± 3.4	4.6 ± 1.4	27.2 ± 5.6
*Galium verum* herb	50% acetone	5.5 ± 1.4	18.3 ± 2.3	2.8 ± 0.3	19.1 ± 3.0
	50% ethanol	5.1 ± 1.4	16.8 ± 2.0	2.6 ± 0.1	22.7 ± 1.6
	80% ethanol	3.5 ± 1.5	11.5 ± 0.6	2.6 ± 0.7	17.4 ± 2.6
*Galium aparine* herb	50% acetone	3.7 ± 0.5	16.0 ± 1.3	2.1 ± 0.2	13.9 ± 1.2
	50% ethanol	4.3 ± 0.9	16.8 ± 1.3	2.1 ± 0.03	20.3 ± 0.4
	80% ethanol	3.8 ± 1.8	12.5 ± 0.7	2.6 ± 0.1	15.2 ± 0.2
*Galium mollugo* herb	50% acetone	7.6 ± 0.3	25.1 ± 0.8	2.9 ± 0.2	20.6 ± 0.7
	50% ethanol	7.1 ± 0.7	23.1 ± 0.5	3.0 ± 0.3	21.8 ± 0.9
	80% ethanol	5.5 ± 1.4	17.6 ± 0.5	3.0 ± 0.2	19.8 ± 3.0

^1^ TE—Trolox equivalent; ^2^ GAE—Gallic acid equivalent; ^3^ QE—Quercetin equivalent; ^4^ AE—Asperuloside equivalent.

**Table 2:** Identification of all quantified compounds in the *Galium* extracts.

No.	Rt, min	Tentative Identification ^1^	Compound Class ^2^	Molecular ion [M − H]^−^ (and MS/MS Fragments)	Appears in the HPLC-DAD-MS/MS Fingerprinting Analysis Chromatograms			
					*Galium verum* Blossoms	*Galium verum* Herb	*Galium aparine* Herb	*Galium mollugo* Herb
**1**	3.92	Deacetylasperulosidic acid	Iridoid glycoside	389.4 (183.2)	+	+	+	+
**2**	4.21	*Unidentified*	Iridoid glycoside	389.5	+	-	-	-
**3**	5.58	Deacetylalpinoside	Iridoid glycoside	373.4 (211.1, 747.6)	-	-	-	+
**4**	5.81	*Unidentified*	Iridoid glycoside	431.5	-	+	-	-
**5**	6.13	*Unidentified*	Iridoid glycoside	707.7	-	-	+	-
**6**	6.66	Neochlorogenic acid	Polyphenol	353.5 (191.5)	-	+	+	+
**7**	7.40	Asperulosidic acid	Iridoid glycoside	431.5 (165.3)	+	+	+	+
**8**	7.91	Quercetin-3-rutinoside-7-glucoside	Flavonoid	771.6 (609.6, 463.4, 301.3)	+	-	-	-
**9**	8.09	Chlorogenic acid	Polyphenol	353.3 (191.0)	+	+	+	+
**10**	8.43	Cryptochlorogenic acid	Polyphenol	353.4 (191.0, 179.0, 173.3, 135.8)	-	-	+	+
**11**	8.61	Asperuloside	Iridoid glycoside	413.4 (191.1, 147.2)	+	+	+	+
**12**	8.82	Isorhamnetin-3-O-rutinoside-7-O-glucoside	Flavonoid	785.6 (623.6, 315.4)	+	-	-	-
**13**	9.29	*Unidentified*	Iridoid glycoside	353.6	-	-	+	-
**14**	11.15	Rutin	Flavonoid	609.5 (463.2, 300.9, 271.9, 255.8, 179.5)	+	+	+	+
**15**	11.71	Quercetin glycoside	Flavonoid	463.6 (301.7, 300.5, 179.7, 151.7)	-	-	+	-
**16**	11.73	Kaempferol-O-glucoside	Flavonoid	447.5 (285.1, 151.7)	+	+	-	+
**17**	12.25	Isorhamnetin-3-O-rutinoside	Flavonoid	623.5 (315.1, 271.7, 255.8)	+	+	+	-
**18**	12.51	Diosmetin isomer	Flavonoid	299.3 (285.1)	-	-	-	+
**19**	12.69	Diosmetin isomer	Flavonoid	299.2 (285.0)	-	-	-	+
**20**	12.82	Dicaffeoylquinic acid isomer	Polyphenol	515.6 (353.5)	+	+	+	-
**21**	13.01	*Unidentified*	Flavonoid	593.9	-	+	-	-
**22**	13.22	*Unidentified*	Polyphenol	516.0	+	+	-	-
**23**	13.47	Diosmetin isomer	Flavonoid	299.6 (284.1)	-	-	-	+
**24**	14.75	Quercetin-3-O-hexose-deoxyhexose	Flavonoid	609.6 (463.4, 301.5)	-	+	-	-
**25**	15.26	*Unidentified*	Flavonoid	623.6	+	-	-	-
**26**	16.45	Quercetin	Flavonoid	301.3 (151.0)	+	+	+	+
**27**	18.72	Kaempferol	Flavonoid	285.3	-	-	-	+
**28**	24.04	Bicalutamide ^3^		429.4	+	+	+	+

^1^ Underlined compounds identified using standard compounds; others based on MS/MS fragmentation data. ^2^ Compound class suggested based on similarities in UV spectra to the standard compounds. ^3^ Internal standard, spiked into all analysed samples at a final concentration of 40 mg/L.

**Table 3:** Quantification of phytochemicals in *Galium* extracts (mg/L).

No.	Rt, min	Standard Compound for Quantification^1^	*Galium verum* Blossoms			*Galium verum* Herb			*Galium aparine* Herb			*Galium mollugo* Herb		
			50% Acetone	50% Ethanol	80% Ethanol	50% Acetone	50% Ethanol	80% Ethanol	50% Acetone	50% Ethanol	80% Ethanol	50% Acetone	50% Ethanol	80% Ethanol
**1**	3.92	Asperuloside	98.23 ± 12.47	215.77 ± 54.79	121.72 ± 22.48	37.37 ± 7.31	149.21 ± 2.67	114.80 ± 19.59	32.85 ± 7.72	178.51 ± 15.60	139.27 ± 4.56	57.76 ± 4.18	315.10 ± 12.92	179.19 ± 46.84
**2**	4.21	Asperuloside	59.33 ± 14.45	130.00 ± 13.30	63.34 ± 7.88									
**3**	5.58	Asperuloside										410.33 ± 28.02	468.27 ± 39.70	332.44 ± 89.06
**4**	5.81	Asperuloside				93.56 ± 12.91	87.41 ± 7.95	76.55 ± 10.46						
**5**	6.13	Asperuloside							50.06 ± 16.65	50.63 ± 13.57	21.28 ± 1.33			
**6**	6.66	Neochlorogenic acid				29.49 ± 0.26	30.53 ± 1.03	27.03 ± 2.37	90.26 ± 30.73	90.94 ± 14.86	45.26 ± 4.22	91.41 ± 1.78	103.56 ± 18.86	69.90 ± 17.19
**7**	7.40	Asperuloside	201.38 ± 23.62	156.67 ± 32.07	118.95 ± 19.98	131.48 ± 7.04	123.32 ± 3.57	115.63 ± 11.42	419.44 ± 161.57	484.60 ± 108.34	246.15 ± 32.36	368.33 ± 27.33	395.70 ± 28.44	250.35 ± 65.31
**8**	7.91	Rutin	124.69 ± 3.20	93.48 ± 14.49	55.06 ± 17.29									
**9**	8.09	Chlorogenic acid	443.70 ± 19.92	347.34 ± 63.75	230.88 ± 54.20	321.72 ± 39.31	287.14 ± 17.18	236.94 ± 25.60	282.20 ± 41.70	300.18 ± 33.30	292.14 ± 14.03	706.44 ± 11.04	710.44 ± 3.96	505.57 ± 151.53
**10**	8.43	Neochlorogenic acid							109.02 ± 9.54	110.66 ± 6.01	109.23 ± 5.37	50.77 ± 3.05	53.86 ± 1.76	33.84 ± 11.44
**11**	8.61	Asperuloside	793.33 ± 17.20	574.46 ± 100.55	565.42 ± 101.40	510.92 ± 51.41	448.61 ± 41.94	476.40 ± 56.23	296.24 ± 115.73	232.80 ± 62.47	394.40 ± 19.17	632.79 ± 27.94	555.91 ± 45.10	548.86 ± 167.57
**12**	8.82	Rutin	44.01 ± 2.65	32.25 ± 8.96	20.63 ± 8.99									
**13**	9.29	Asperuloside							54.03 ± 5.98	47.85 ± 0.85	44.54 ± 3.60			
**14**	11.15	Rutin	618.22 ± 9.44	456.93 ± 35.66	383.91 ± 91.16	118.41 ± 16.04	104.94 ± 7.00	92.46 ± 16.69	299.96 ± 8.95	290.76 ± 17.52	253.78 ± 11.26	59.10 ± 2.24	63.68 ± 3.88	59.75 ± 10.64
**15**	11.71	Rutin							4.75 ± 0.47	5.22 ± 1.24	4.56 ± 1.02			
**16**	11.73	Rutin	347.04 ± 1.91	252.78 ± 30.68	219.79 ± 47.19	108.91 ± 15.60	96.25 ± 9.46	78.25 ± 10.16				56.50 ± 3.46	59.89 ± 1.78	54.01 ± 15.42
**17**	12.25	Rutin	110.30 ± 3.33	87.59 ± 14.55	66.13 ± 15.24	22.77 ± 2.81	21.78 ± 1.97	17.02 ± 2.88	87.01 ± 9.20	88.90 ± 2.76	75.26 ± 6.96			
**18**	12.51	Rutin										241.83 ± 2.73	249.46 ± 10.03	191.77 ± 50.62
**19**	12.69	Rutin										465.66 ± 11.13	675.07 ± 358.40	377.44 ± 66.10
**20**	12.82	Neochlorogenic acid	87.55 ± 9.43	66.12 ± 11.13	52.34 ± 12.29	61.22 ± 9.30	50.37 ± 7.63	44.14 ± 5.26	19.20 ± 3.56	18.96 ± 3.58	19.85 ± 1.81			
**21**	13.01	Rutin				5.72 ± 1.40	4.95 ± 1.23	2.84 ± 1.23						
**22**	13.22	Neochlorogenic acid	482.16 ± 32.26	334.60 ± 31.88	301.17 ± 78.84	27.88 ± 3.44	24.29 ± 3.05	18.01 ± 3.62						
**23**	13.47	Rutin										73.50 ± 3.40	75.99 ± 5.10	66.31 ± 16.64
**24**	14.75	Quercetin				9.54 ± 0.46	9.07 ± 0.20	8.82 ± 0.47						
**25**	15.26	Quercetin	11.19 ± 0.22	9.81 ± 0.53	8.88 ± 0.87									
**26**	16.45	Quercetin	10.11 ± 0.37	9.36 ± 0.50	7.83 ± 0.74	8.88 ± 0.57	8.49 ± 0.61	9.76 ± 0.33	4.47 ± 1.54	6.08 ± 0.71	3.02 ± 0.29	6.42 ± 0.22	6.81 ± 0.45	6.05 ± 0.19
**27**	18.72	Quercetin										10.66 ± 0.48	11.37 ± 0.60	8.49 ± 0.58

^1^ Underlined compounds identified using standard compounds; others quantified using calibration curves of standard compounds with the most similar UV spectrum. All UV spectra (200–400 nm) are provided in the Supplementary Materials (Figures S2–S29), as measured from the analyses of 50% ethanol extracts.

**Table 4:** Identified volatile compounds in the samples of the *Galium* species.

Compound	Rt, min	*Galium verum* Blossoms, %	*Galium verum* Herb, %	*Galium aparine* Herb, %	*Galium mollugo* Herb, %
*β*-Methylbutanal	3.72	-	-	1.7	<1.0
2-Methyl-4-hydroxy-cyclobutanone	3.84	-	-	2.2	-
*α*-Methylpropanoic acid	5.37	-	-	2.0	-
Hexanal	6.44	1.2	1.9	2.4	1.1
***β***-Methylbutyric acid	7.43	<1.0	-	7.5	2.8
α-Methylbutyric acid	7.72	<1.0	<1.0	3.1	-
(*E*)-2-Hexenal	7.85	-	-	-	2.7
2,5,5-Trimethyl-1,3,6-heptatriene	9.98	1.8	<1.0	-	-
*β*-Terpinen	11.38	-	4.0	-	-
Hexanoic acid	11.41	<1.0	-	-	3.4
*β*-Pinene	11.49	-	7.7	-	-
4-Terpinenyl acetate	12.22	1.3	-	-	-
d-Limonene	13.04	-	1.0	-	-
Eucalyptol	13.12	3.4	4.8	-	-
Salicylaldehyde	13.47	4.3	<1.0	2.1	1.4
Artemisia ketone	13.96	15.2	<1.0	-	1.6
Artemisia alcohol	14.66	4.5	<1.0	-	-
2-Methylene-hexanal	14.83	-	1.2	-	-
Linalool	15.10	-	2.0	-	-
Nonanal	15.21	2.9	<1.0	4.1	5.6
*δ*-Thujone	15.67	7.2	<1.0	-	1.2
Camphor	16.50	2.2	11.1	-	-
*p*-Menthone	16.75	<1.0	7.0	4.4	<1.0
Artemisyl acetate	17.17	13.5	<1.0	-	2.4
*α*-Terpineol	17.80	-	1.1	-	-
Methyl salicylate	17.94	1.0	<1.0	-	-
Estragole	17.99	-	2.3	-	-
Thymol methyl ether	18.96	5.5	<1.0	3.8	9.2
Carvone	19.29	<1.0	4.4	-	1.1
Anethole	20.40	1.1	4.8	31.4	11.9
Thymol	20.49	<1.0	-	8.4	6.1
Isomenthyl acetate	20.59	-	1	-	-
2,3,5,8-Tetramethyl-decane	20.67	-	1.1	-	-
2-Ethyl-4,5-dimethyl-phenol	20.77	-	-	<1.0	1.5
Copaene	22.84	-	2.9	-	-
*β*-Bourbonene	23.11	<1.0	<1.0	<1.0	1.0
Helminthogermacrene	23.25	1.3	1.2	-	<1.0
Bornyl isobutyrate	23.79	1.5	-	-	-
***β***-Caryophyllene	24.00	3.6	3.1	3.0	10.0
Calarene	24.30	-	1.4	-	-
*α*-Caryophyllene	24.82	<1.0	1.0	<1.0	-
*γ*-Muurolene	25.32	<1.0	1.0	-	-
*α*-Curcumene	25.40	<1.0	3.1	-	-
*β*-Cubebene	25.49	10.6	<1.0	-	-
Germacrene D	25.50	<1.0	1.6	-	13.4
*β*-Acorenol	25.64	<1.0	1.2	<1.0	2.6
Aciphyllene	25.82	-	1.1	-	-
Isobisabolene	26.04	5.3	1.0	<1.0	2.8
Bornyl pentanoate	26.24	1	-	-	-
Dihydroactinidiolide	26.67	-	2.5	8.5	10.2
Caryophyllene oxide	27.90	-	1.5	-	2.0
Hexahydropseudoionone	33.22	-	<1.0	8.1	5.1

## Data Availability

The data presented in this study are available on request from the corresponding author.

## Supplementary Materials

The following supporting information can be downloaded at: https://www.mdpi.com/article/10.3390/molecules28062867/s1. The graph illustrating the correlation of polyphenolic content and antioxidativity of the extracts (Figure S1) as well as the UV spectra of all quantified phytochemicals measured from the analyses of 50% ethanol extracts (Figures S2–S29) are provided as a separate file.

## Abbreviations

AE—asperuloside equivalent; DAD—diode array detector; DVB—divinylbenzene; FID—flame ionisation detector; GAE—gallic acid equivalent; GC—gas chromatography; HPLC—high performance liquid chromatography; MS—mass spectrometry; NMR—nuclear magnetic resonance; ORAC—oxygen radical absorbance capacity; PDMS—polydimethylsiloxane; QE—quercetin equivalent; SPME—solid phase microextraction; TE—Trolox equivalent; UHPLC—ultra-high performance liquid chromatography; UV—ultraviolet (radiation).
