# Supplementary material for: Phytochemical Screening and Antioxidant Activity of Selected Estonian Galium Species
Source: Molecules. 2023 Mar 22;28(6):2867. doi: 10.3390/molecules28062867 (PMC10056973; doi:10.3390/molecules28062867)
Supplement: Supplementary file 1 [file molecules-28-02867-s001.zip › molecules-2271639-supplementary.pdf]

Phytochemical Screening and Antioxidant Activity of Selected Estonian *Galium* Species

Pille-Riin Laanet [1,*], Piret Saar-Reismaa[1,2], Piia Jõul[1], Olga Bragina[1,3] and Merike Vaher[1]

[1] Department of Chemistry and Biotechnology, Tallinn University of Technology, Akadeemia tee 15, 12618 Tallinn, Estonia

[2] HAN BioCentre, Laan van Scheut 2, 6525 EM Nijmegen, the Netherlands

[3] National Institute for Health Development, Hiiu 42, 11619 Tallinn, Estonia

* Correspondence: pille-riin.laanet@taltech.ee; Tel.: +372 620 4325

Supplementary Materials

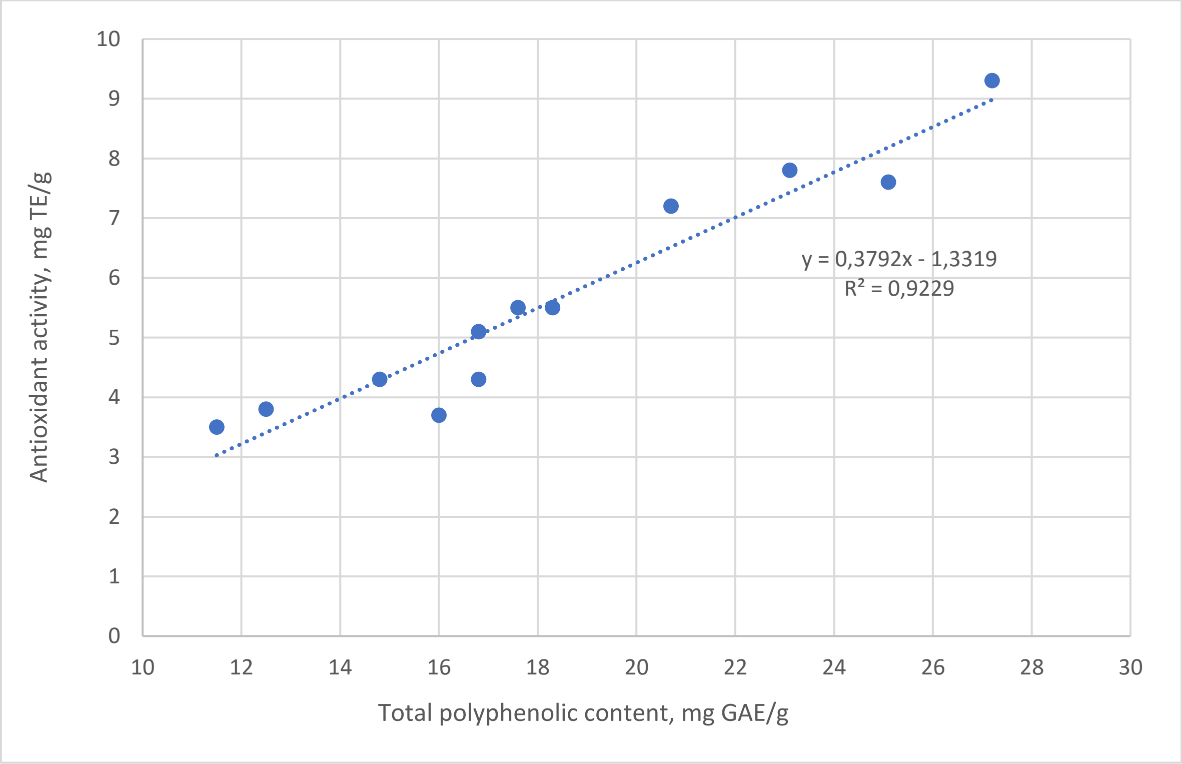

Figure S1. The correlation of phenolic content and antioxidant properties of the *Galium* extracts

Figures S2–S29. UV spectra of all quantified compounds, measured from the analyses of 50% ethanol extracts in the range of 200 to 400 nm (compound numeration as per Tables 2 and 3):

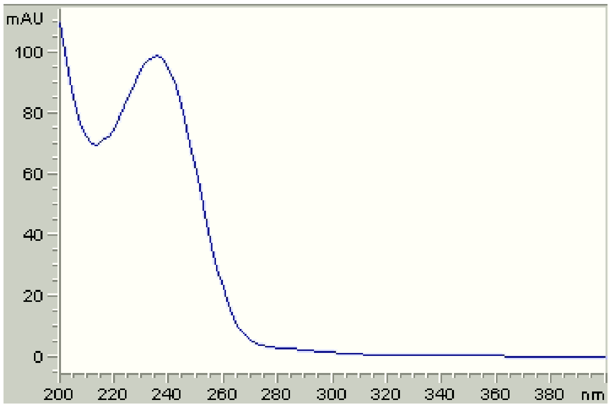

Figure S2. UV spectrum of compound **1** (quantified using **asperuloside calibration**)

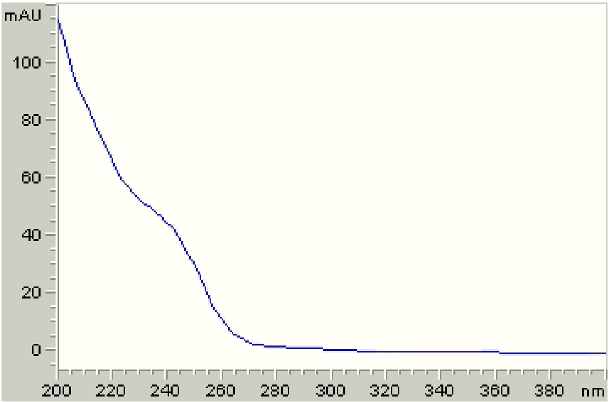

Figure S3. UV spectrum of compound **2** (quantified using **asperuloside calibration**)

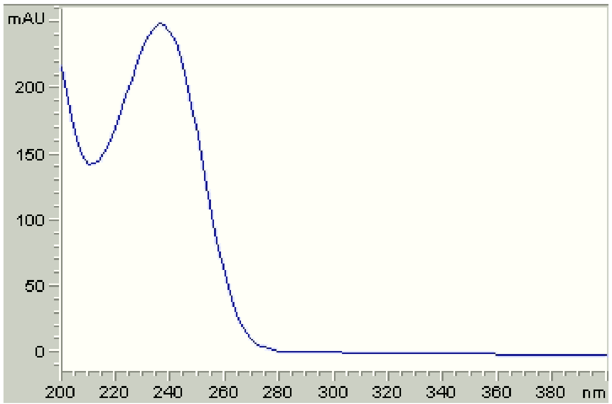

Figure S4. UV spectrum of compound **3** (quantified using **asperuloside calibration**)

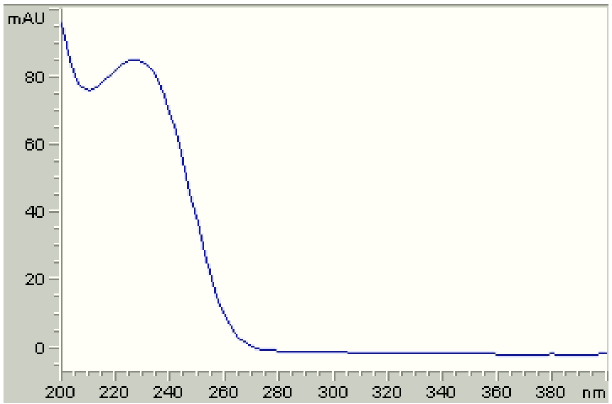

Figure S5. UV spectrum of compound **4** (quantified using **asperuloside calibration**)

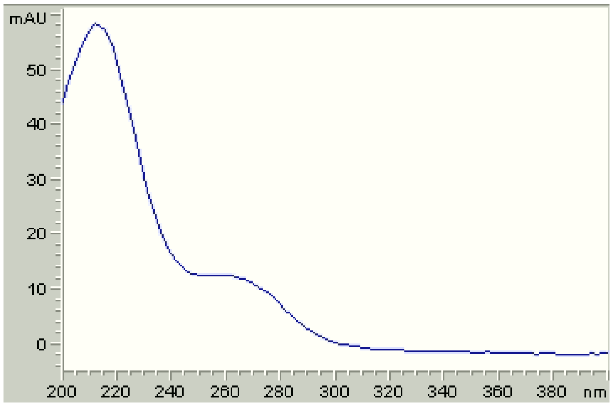

Figure S6. UV spectrum of compound **5** (quantified using **asperuloside calibration**)

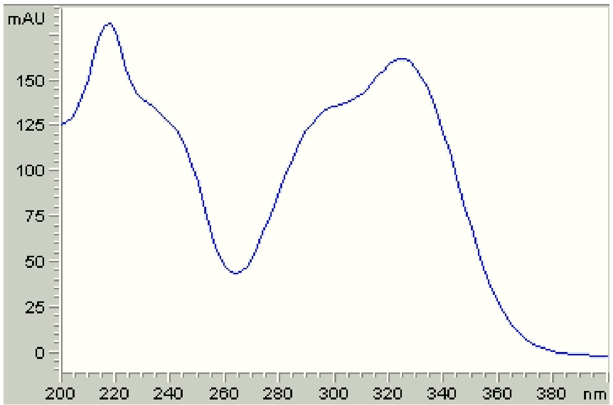

Figure S7. UV spectrum of compound **6** (identified as **neochlorogenic acid** using a standard compound)

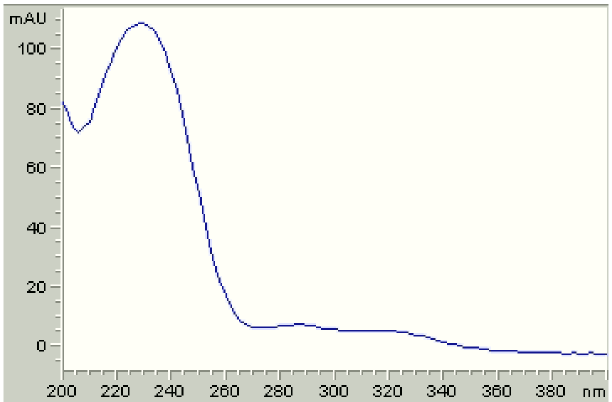

Figure S8. UV spectrum of compound **7** (quantified using **asperuloside calibration**)

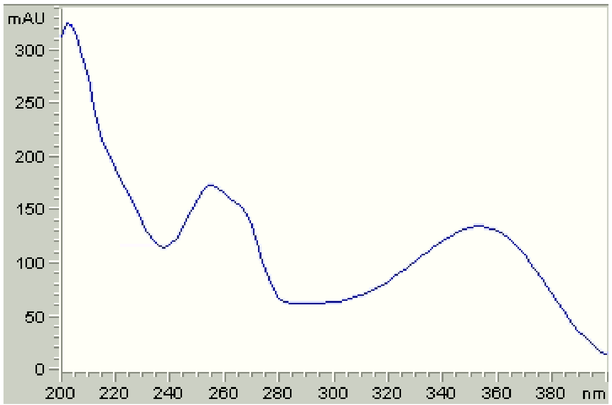

Figure S9. UV spectrum of compound **8** (quantified using **rutin** calibration)

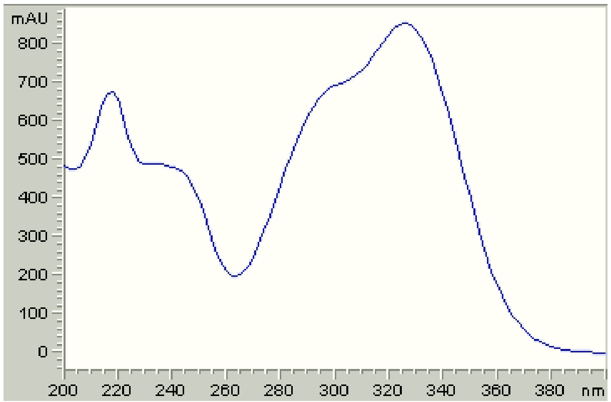

Figure S10. UV spectrum of compound **9** (identified as **chlorogenic acid** using a standard compound)

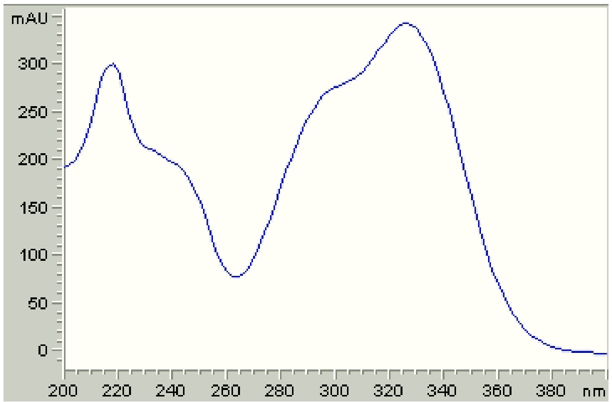

Figure S11. UV spectrum of compound **10** (quantified using **neochlorogenic acid calibration**)

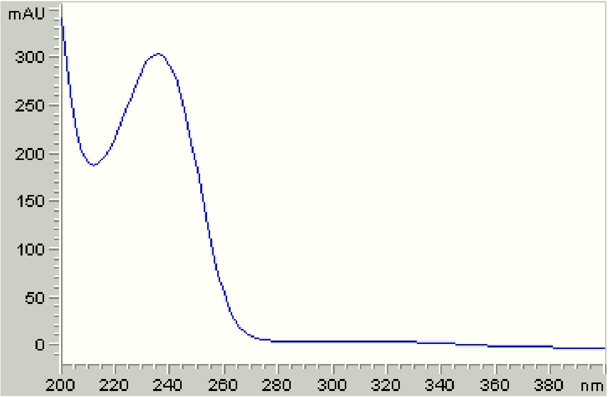

Figure S12. UV spectrum of compound **11** (identified as **asperuloside** using a standard compound)

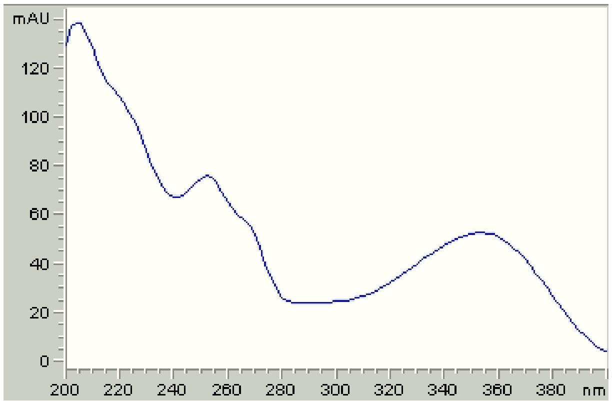

Figure S13. UV spectrum of compound **12** (quantified using **rutin calibration**)

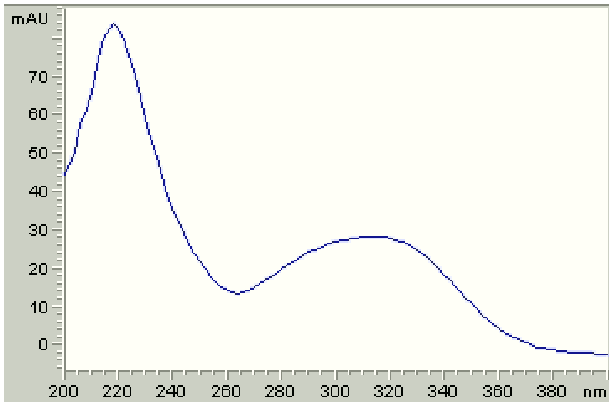

Figure S14. UV spectrum of compound **13** (quantified using **asperuloside calibration**)

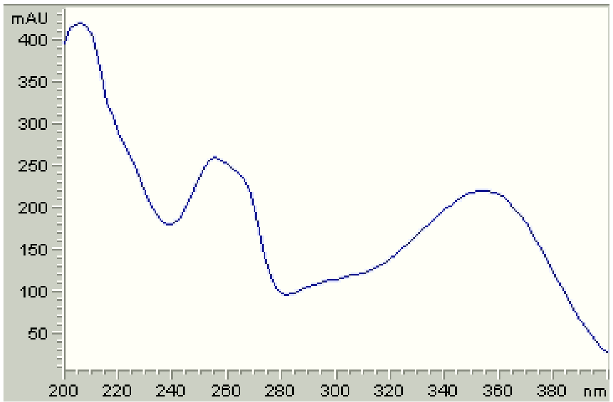

Figure S15. UV spectrum of compound **14** (identified as **rutin** using a standard compound)

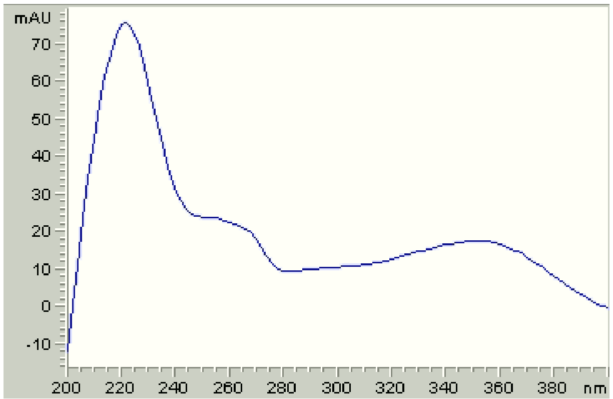

Figure S16. UV spectrum of compound **15** (quantified using **rutin calibration**)

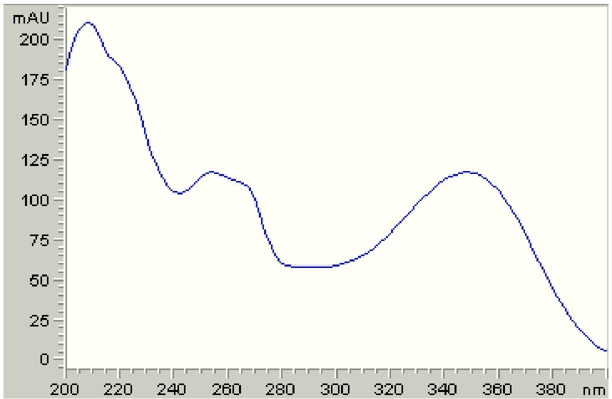

Figure S17. UV spectrum of compound **16** (quantified using **rutin calibration**)

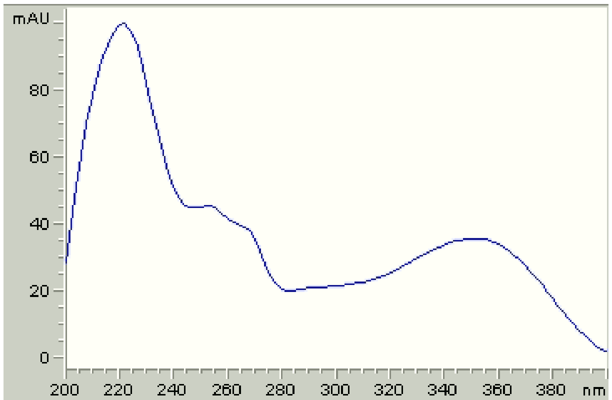

Figure S18. UV spectrum of compound **17** (quantified using **rutin calibration**)

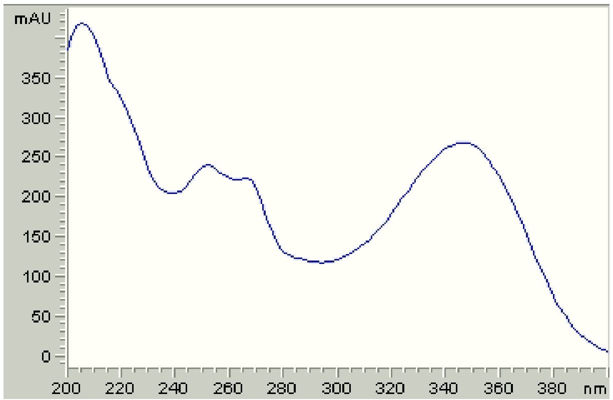

Figure S19. UV spectrum of compound **18** (quantified using **rutin calibration**)

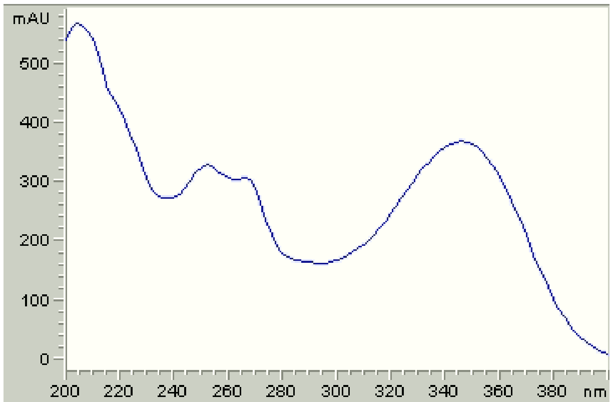

Figure S20. UV spectrum of compound **19** (quantified using **rutin calibration**)

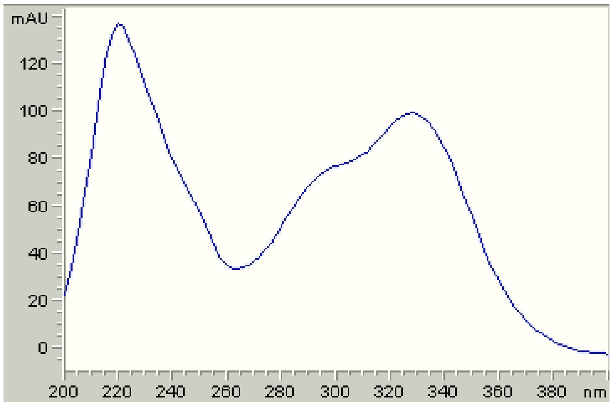

Figure S21. UV spectrum of compound **20** (quantified using **neochlorogenic acid calibration**)

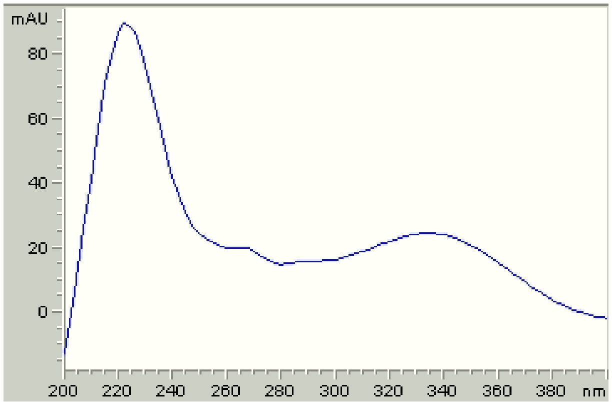

Figure S22. UV spectrum of compound **21** (quantified using **rutin calibration**)

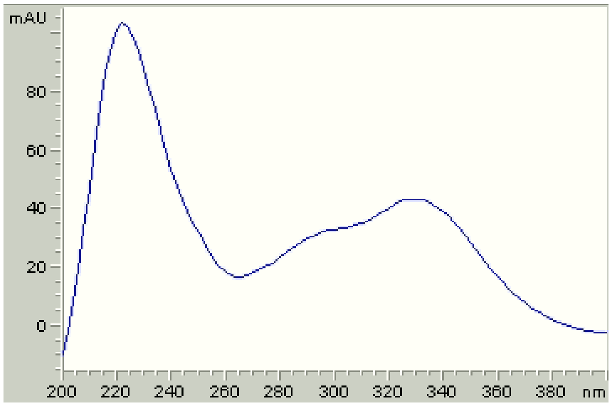

Figure S23. UV spectrum of compound **22** (quantified using **neochlorogenic acid calibration**)

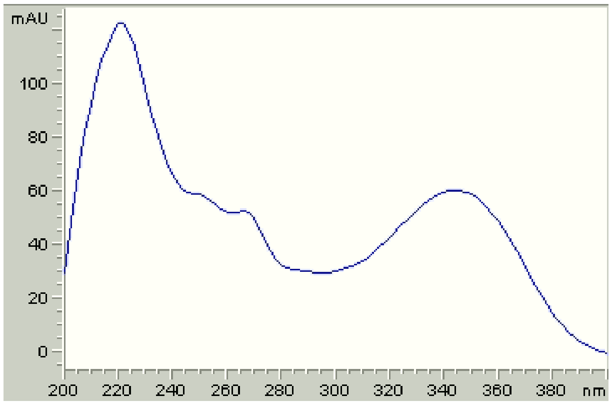

Figure S24. UV spectrum of compound **23** (quantified using **rutin calibration**)

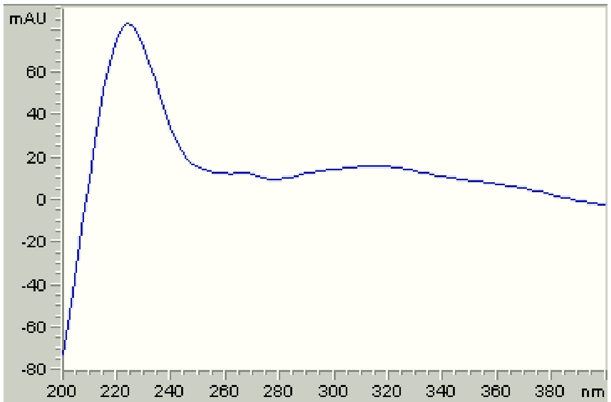

Figure S25. UV spectrum of compound **24** (quantified using **quercetin calibration**)

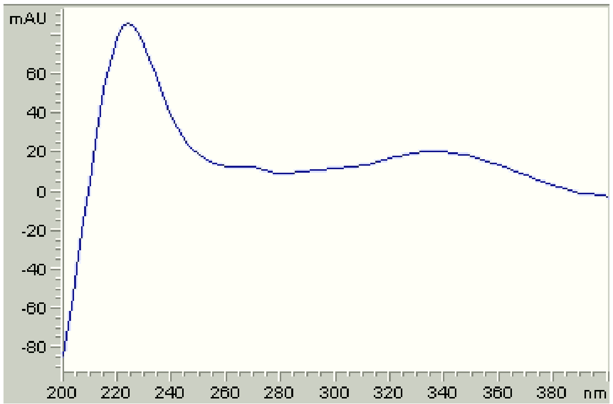

Figure S26. UV spectrum of compound **25** (quantified using **quercetin calibration**)

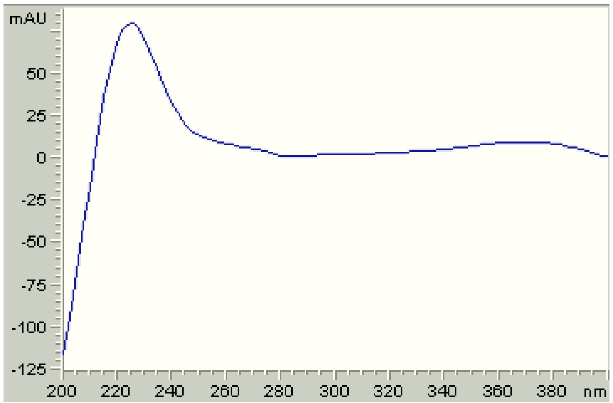

Figure S27. UV spectrum of compound **26** (identified as **quercetin** using a standard compound)

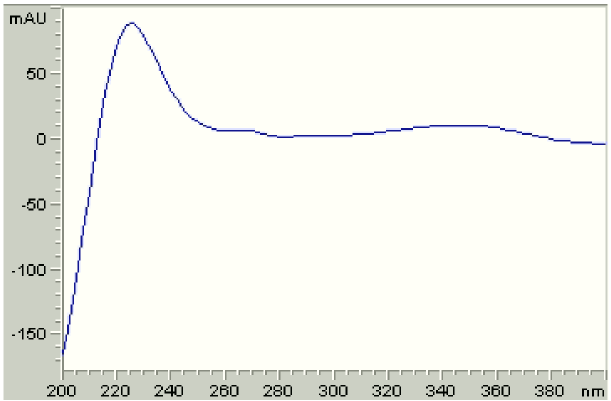

Figure S28. UV spectrum of compound **27** (quantified using **quercetin calibration**)

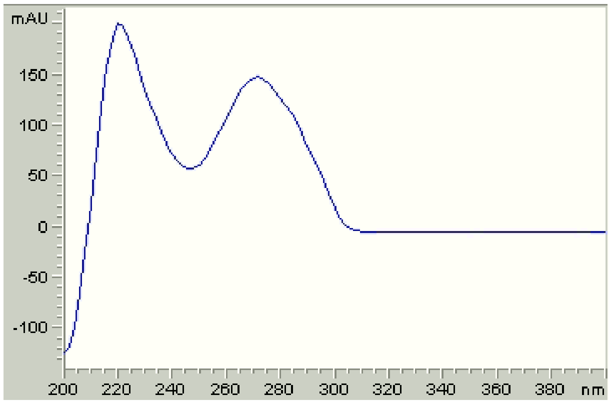

Figure S29. UV spectrum of compound **28** (internal standard **bicalutamide**)
